# Management of diabetes and diabetes policies in Turkey

**DOI:** 10.1186/1744-8603-9-16

**Published:** 2013-04-18

**Authors:** Mehtap Tatar

**Affiliations:** 1 Department of Healthcare Management, Faculty of Economics and Administrative Sciences, Hacettepe University, Beytepe 06800, Ankara, Turkey

**Keywords:** Diabetes care, Turkey, Turkish healthcare policy

## Abstract

**Background:**

Diabetes and its complications are among the present and future challenges of the Turkish health care system. The objective of this paper is to discuss the current situation of diabetes and its management in Turkey with special emphasis on the changing policy environment.

**Methods:**

A literature review in databases such as PUBMED was performed from 2000 to 2011. This synthesis was complemented by grey literature, personal communication and contact with national and provincial health authorities and experts in diabetes from Turkey.

**Results:**

The literature review and expert consultations indicated a growing policy emphasis on diabetes. Both the public and private sectors, non-governmental organizations have initiated policy papers to shape the outlook of diabetes care in the future. This is in line with the current dynamics of the healthcare system.

**Conclusions:**

Diabetes care will be high on the agenda in future. Evidence based policy-making is the key to implement the policies adopted so far and a supportive environment is needed.

## Introduction and objectives

Turkey has been undergoing a radical reform process since the beginning of the Health Transformation Program (HTP) in 2003 [1]. The major focus areas of this transformation have been on the purchaser provider split, reorganizing the primary and secondary level of care and the introduction of a general health insurance scheme.

Before 2003, Turkey had a very fragmented health care system with large inequalities among different segments of the population. Accessibility, equality, quality and efficiency problems were the main concerns of the Turkish healthcare system before 2003. The government addressed the inequality problem by merging all existing public financing schemes under the Social Security Institution with gradual equalization of health benefits packages of these schemes. The monopsonic power of the SSI as the main purchaser of health care services both from the public and private sector has also shifted the balance of health policy-making power from the Ministry of Health (MoH) to the organization. The reimbursement rules applied by the organization shape not only the financing but also provision of health care services as well.

Major policy changes were introduced in the provision of health care services as well. As in the case of financing, health care services were also very fragmented and access was restricted for the majority of the population before 2003. First, health care facilities of other organizations with the exception of the university and the Ministry of Defense facilities were transferred to the MoH. Second, a family practitioner scheme was introduced that changed the organization of the primary level of care. Although there is not a compulsory referral system yet, family practitioners play an important role in improving access to the health care system. A number of other changes supporting the transformation of the system were also made such as introduction of a performance based payment system, changes in the reimbursement and pricing policies and others. A detailed analysis of the current health care system can be found in Tatar et al. [2].

Although a comprehensive impact assessment of these reforms has not been made yet, improvements in selected indicators confirm the analysis made here. The main health indicators used to assess the health status level of a country have improved considerably after 2000. Although some part of this improvement is attributable to the advances in the socioeconomic indicators as well, the improvements especially in accessibility figures show that the HTP had a positive influence as well. For instance, the infant mortality rate has decreased from 31.4 in 2002 to 7.7 in 2011 [3]. Life expectancy has also increased from 70  in 2000 to 75 in 2011 [3]. In line with these improvements, the maternal mortality rate has also declined in the past years and according to the World Health Organization, Turkey is among 14 countries which achieved more than a 5.5% yearly decline in maternal mortality [4]. As far as the main causes of death are concerned, Turkey has a list of diseases similar to those from developed countries. The Burden of Disease (BoD) study concluded in 2003 before the implementation of HTP and showed that ischemic heart disease and cerebrovascular diseases were among the highest causes of deaths with 21.7% and 15% shares in total deaths respectively. Diabetes Mellitus was ranked 8^th^ on the list with a 2.2% share in total deaths [5]. A more recent study conducted by the Turkish Statistics Institute listed the top five causes of death as circulatory system 39,9%, malignant neoplasms 20,7%, diseases of the respiratory system 8,9%, Endocrine, nutritional and metabolic diseases 6,4% and external causes of injury and poisoning 4% [6].

The changing environment in the Turkish healthcare system has also changed the approach to certain diseases including diabetes. The objective of this paper is to discuss diabetes and its management in Turkey with special emphasis on the changing policy environment and its impact on health promotion policies in general and diabetes in particular.

## Methodology

A literature review in PUBMED was performed from 2000 to 2011. This synthesis was complemented with grey literature, personal communication and contact with national and provincial health authorities and experts in diabetes from Turkey. The literature review was carried out with key words ‘diabetes-cost-Turkey’ and ‘diabetes-policy-Turkey’. Total 37 studies for the former and 15 studies for the latter search was found. None of the studies were directly related to the topic searched and diabetes was in general taken in the sub-group analysis of the study population. Experts in the MoH and SSI were also consulted to explore the unpublished data and policies. National policy documents and all related documents from public and private stakeholders including non-governmental organizations were also the main sources of information.

The literature review revealed that both national and international publications focused on the risk factors of diabetes in certain sub-populations, treatment methods of diabetes with special emphasis on complications, prevalence of disease in small geographical areas and few studies on quality of life measurements of diabetes patients. The majority of these studies covered a small sub-population or geographical area, making it impossible to infer national figures.

## Results

Turkey does not have an official diabetes registry and international and national surveys are the only data sources for the burden of diabetes. Currently, a database study named Health-Net is being developed under the second phase of HTP. The Hospital Information System and Family Practitioner Information System collect data on diagnosis date, patient’s height, weight, waist measurement, exercise status, compliance to diet, blood pressure, thyroid examination and co-morbidity. However, the lack of a referral system is a barrier to having aggregate data on all health indicators (including diabetes) as patients can visit any facility any time without a referral from a lower level.

### Prevalence

According to the 2011 Diabetes Atlas of the International Diabetes Association (IDF), the adult population between the ages of 20–79 was 47.322.400 in 2011 and diabetes cases among this population totalled 3.502.270. Diabetes is more prevalent among females representing 58.1% of total diabetes cases. According to the estimates of the IDF, 1.256.440 people have undiagnosed diabetes. This figure addresses a considerable unmet need in diagnosis and treatment of diabetes in the country. Estimated prevalence of diabetes was reported as 7.4% and impaired glucose tolerance was estimated at 6.7%. The number of diabetes related deaths was estimated at 31.931 in the same report [7].

Four national surveys provide evidence about the burden of diabetes in Turkey: The National Burden of Disease and Cost Effectiveness Study [5]; The Turkish Diabetes Epidemiology Study I [8] and II [9] (TURDEP I, TURDEP II) and TEKHARF (The Turkish Adult Risk Factor Survey) [10]. As stated earlier, in the burden of disease study (BoD), diabetes was ranked 8^th^ on the list of causes of death with 2.2% share among total deaths in the country. Diabetes constituted 1.9% of the total Disability Adjusted Years (DALYs) and the figures were 1.64% for total Years of Life Lost and 2.17% for total Years Lost with Disability. The BoD study found the annual incidence of diabetes as 3.820 per 100,000 population (3.820 for males and 3.210 for females). The study concluded that the prevalence of the disease was 6% [5].

TURDEP I and TURDEP II provide the most detailed analysis of the epidemiology of diabetes in Turkey and also allow comparison of figures between 1997–2010. TURDEP I was conducted between September 1997 to March 1997 in 540 health centres across the nation. 29,050 eligible people from these centres were invited to the survey and 24,788 completed the study. In order to represent the age and gender structure at the regional level, people aged 20 and over and living in defined settlements were invited to the study

Blood tests and other measurements were taken together with a questionnaire to assess socioeconomic characteristics [8]. TURDEP II [9] was conducted from January to June 2010 in the same 540 centres with the same methodology to allow for comparison of results. 26,499 people were invited to take part in the study from these centres and 92% of the invitees participated in the research. Table 1 below shows the results of the two surveys.

**Table 1:** Results of TURDEP I and II

	**TURDEP I**	**TURDEP II**
	**1997**	**2010**
Study population ≥ 20 year?	24 788	26 499
Mean age	41 (SD 14.7)	45.8 (SD 15.4)
BMA≥30 kg/m2	22%	35.9%
High blood pressure prevalence	29%	31.3%
National prevalence	7.2%	13.7%
Impaired glucose tolerance	6.7%	6.3%
Those who aren’t aware of having the disease	32%	45%
Undiagnosed		

[8,9].

TURDEP II study also revealed that measurements reflecting the risk factors have increased from the first study conducted 13 years earlier. For instance, weight for females increased 8 kg since the first study and this figure was 6 kg for males. Similar increases were also observed in waist and hip measurements as well (waist measures increased 6 cm for males, 7 cm for females; hip measures increased 2 cm for males and 7 cm for females). According to the study, diagnosis age of diabetes was five years earlier than the TURDEP I Study. The study concluded that diabetes incidence has increased 90% and obesity has increased 44% between the two studies.

The last national survey providing data on diabetes was the TEKHARF This study was aimed at assessing the cardiovascular health of the population and included diabetes as a risk factor. In the study, 3401 people were screened in terms of diabetes between 1997/98 and 2004/2005. The study concluded that the incidence of diabetes for those 35 and over reached 360,000 (1.22%) in 2008 [10].

### Costs

Cost of disease studies are not very common in Turkey and diabetes is not an exception. There are three sources of information with methodological limitations. The first one was undertaken in 1998 with the aim of estimating the direct medical costs of diabetes in an adult population. The study covered 959 patients enrolled in the study for two months from 13 centres at the tertiary level. Those included in the study had high systemic complication rates and poor glycemic control. The study concluded that the annual direct cost of diabetes treatment per patient was USD$ 1,100-2,100 and half of this cost was due to the treatment cost of the disease. The cost for patients with complications of diabetes was three times higher compared to the patients without complications. The direct costs increased 6.5 fold in case of hospitalization. As the study was undertaken in tertiary facilities, the complication rates may not represent the complication rate in the Turkish population. In order to take this into account, the researchers used a range of 0-70% complication rate in their model and estimated the overall annual direct cost of diabetes in Turkey as USD$ 1–2.5 billion. They also concluded that a 10% increase in the complication rate added a cost of USD$ 180–190 million to the overall cost of diabetes [11].

The second study regarding cost of diabetes screening and treatment is the Burden of Disease and Cost Effectiveness Study [5]. In the study, the incidence of diabetes was estimated as 38.20 per thousand (prevalence of 5.6%). Based on this assumption along with assumptions regarding the effectiveness of the interventions, it was concluded that population based screening and treatment may prevent 828,835 cases per year in the long run. The study’s estimates for diabetes treatment are presented in Table 2.

**Table 2:** Cost of diabetes treatment in Turkey (Results of the Burden of Disease Study)

	**Health centre based non-insulin dependent diabetes treatment (100% cases treated in health centres)**	**Hospital based treatment for insulin dependent diabetes (100% cases treated in hospitals)**	**Hospital based treatment for non-insulin dependent diabetes (100% cases treated in hospitals)**
Cases to be treated	3 405 113	378 346	3 405 113
Incidence cases	2 331 099	259 011	2 331 099
No of deaths	8 594	955	8 594
DALYs saved	2 258 798	470 474	4 234 269
Cost/Case (USD$)	176.22	1 580.70	448.62
Cost/Case (USD$ PPP)	460.98	4 135.12	1 173.59
Total cost (USD$)	480 039 240.45	478 441 207.31	1 222 081 512.03
Cost per DALY (USD$)	222.36	1 016.93	288.62

[5].

The monitoring and treatment of diabetes had the highest cost effectiveness ratio when compared with other public health interventions. The high cost-effectiveness ratio was attributed to the chronic character of the disease with high prevalence and relatively high cost of medical management of the disease. Although the cost of intervention is high, the cost effectiveness ratio was found as USD$ 1,097 per DALY, less than the GDP per capita of the time.

### Costs of complications

The third study for the cost of diabetes treatment was carried out from January 1- December 31 2009 to determine the direct costs of cardiovascular, ophthalmological, neurological and nephrological complications in patients with diabetes. The study covered the real data of 7,095 patients in that period and restricted results were shared in a national diabetes congress. As there is not any information on the methodology of the survey currently, it is not possible to comment on the quality of the conclusions reached. The study concluded that the share of cardiovascular complications in the total cost of diabetes was 32.6%. The figure was 25% for nephrological complications, 6.4% for eye complications and 6% for neurological complications. The study also concluded that the pharmaceutical expenditures for diabetes treatment constituted only 10.9% of the annual direct cost of the disease to the SSI. Diabetes expenditures are not seen as a separate item in the health care budget as disease specific budgeting is not done in Turkey. Therefore the total amount of resources allocated to diabetes cannot be estimated very easily.

In terms of comparison with other high incidence and high mortality illnesses, diabetes patients are treated equally in terms of diagnosis and treatment opportunities. Diabetes patients are exempt from co-payments for drugs if they are prescribed following the rules listed below. There are also co-payments for visits to the health care facilities. These co-payments increase from primary to secondary and tertiary care and are highest in case of private facilities. There are no exemptions from these co-payments.

In terms of availability of medicines, there isn’t a threat to the patients’ health, in other words, the required medicines at all stages of the treatment of the disease are available in Turkey. However, as part of the cost containment measures for all pharmaceuticals, in the last two years the marketing and reimbursement procedures for new molecules have slowed down. Turkey uses both external and internal reference pricing and public discounts for reimbursement of drugs. After getting a marketing approval from the MoH, the lowest price in France Greece, Italy, Spain and Portugal determines the price of the original drug is priced with. Once a generic enters the market, the generic is priced at 60% of the reference price and the price of the original also decreases automatically to that level as well. The number of generics in the market does not change these rates. After getting a market price for an original medicine, the firm applies to the SSI for reimbursement with a dossier covering effectiveness and safety date and pharmacoeconomic analysis for the product. All original drugs have to give 41% discount from the market price in order to get a reimbursement approval.

The reimbursement agency can put restrictions on the use of the medicine by enforcing rules. Turkey also has an internal reference pricing system where all medicines are clustered in drug equivalent groups and the SSI reimburses the cheapest price plus 10% in each group. If the prescribed drug is over this band price, then the patient has to pay the difference or the pharmacist substitutes the prescribed medicine with another one within the band limits. The pricing and reimbursement environment has started to negatively impact the healthcare environment as multinational companies have started to re-evaluate their marketing strategies.

## Discussion

The research outlined above reveals that the prevalence of diabetes has doubled and the burden of the disease on the Turkish healthcare system has increased. Multiple factors may have played a role in the upsurge of the disease. First, the accessibility to the health care system has improved considerably in the last decade and the probability of being diagnosed with the disease has also increased. Second, as a part of the transformation program, primary care services have also improved considerably and after the introduction of the family practitioner scheme diagnosis of diabetes at later stages may have also improved. Third, life-style related risks are increasing in Turkey as the increases in obesity rates etc. shows.

The International Diabetes Association has made some estimates about the health care expenditures for the treatment of diabetes as well. For the year 2010, the mean diabetes related expenditure per person with diabetes was calculated at USD$ 933. The number of people with diabetes between 20–79 was estimated as 3,502,270 for the same year [11]. According to these estimates, the figure for total diabetes expenditure for 2010 was more than USD$ 3 billion.

There is a growing need to study the cost of diabetes and its share in total health care expenditures in Turkey. This issue was tackled in the National Action Plan for diabetes as well. One of the targets of the Plan was to undertake regular cost of disease studies to support effective and efficient management of the disease. The following actions were listed to achieve the target [12]:

•Cost packages in line with the MoH diabetes care standards would be prepared;

•Actions would be taken to enforce the MoH diabetes standards for reimbursement decisions; and

•Actions would be taken to reimburse all activities related to diabetes including education for diet, physical activity and exercise.

Similar recommendations were also made in the Diabetes 2020 project as well. Cost of disease studies and health technology assessments were proposed with special emphasis on the societal perspective. These recent initiatives support the claims that there will be more emphasis on the cost of diabetes in the Turkish health care system.

The introduction of health promotion policies within the overall context of health care policies gained pace after 2008. As life style related chronic diseases started to occupy the agenda, policies related to obesity, diabetes, and high blood pressure were accepted as priority areas to focus on.

The MoH declared its first National Diabetes Program in 1994 in line with the strategies declared in ‘St. Vincent Declaration’. After the declaration of the program, diabetes polyclinics and centres were established in 15 provinces. The program was revised as ‘National Diabetes, Obesity and Hypertension Program’ in 1996. This program can be regarded as a pioneer of the following policy initiatives. As similar initiatives in the 1990s the program did not meet the pre-determined objectives.

In 2009 the initiation of the project ‘Diabetes 2020’ attracted the attention of different stakeholders to the disease once again. The project supported by the MoH, the World Health Organization and the IDF, was also a very good example of inter-sectoral action in health care in Turkey. Apart from the MoH and international partners, a wide range of stakeholders from the non-governmental organizations including patients, academicians and providers were involved in the project. In 2009, a comprehensive report on the issues discussed in the diabetes profile workshop was published [13]. The report analyzed the current situation in diabetes from a broad perspective and covered topics from the prevention of diabetes and diabetes-related complications to the coordination, organization and planning of diabetes care, the financial and economic aspects of diabetes, and the need for an information system for diabetes and a national registry. The results of the second workshop that focused on the solutions to the current problems were published in 2010 [14].

The MoH, in line with its increasing emphasis on life style policies and chronic diseases, launched the Turkish Diabetes Prevention and Control Program in 2009. This program also involved a large number of participants from governmental organizations, non-governmental organizations, academicians, patient groups and health care providers. In 2011, The Action Plan for 2011–2014 was published [12]. The objectives of the program were set out as follows:

•Increasing awareness for diabetes and risk factors;

•Promoting public adoption of healthy life styles;

•Controlling diabetes through early diagnosis;

•Improving treatment and monitoring of diabetes in line with current standards; and

•Decreasing diabetes-related complications.

In the Action Plan, targets for 2014 were determined, followed by actions to be taken to achieve these targets. The preparation of a national diagnosis and treatment guideline for diabetes and its complications was stated as the most critical step. According to the Plan, the MoH Diabetes Standards determining the treatment algorithm for the disease will be developed as the first initiative of the program. This disease specific guideline by the MoH will be its first example in the Turkish health care system and will be the basis for reimbursement of prevention, treatment and monitoring of the disease. Indicator based targets are also determined in the Action Plan. Some of these are as follows:

•The incidence, prevalence and impaired glucose tolerance will be reduced by 5% by 2020.

•At least 10% of the general population will be educated on diabetes in the first 5 years.

•The percentage of patients not aware of the disease will be decreased by 10% in 5 years.

•Periodical cost analysis reports of the disease will start to be published at the end of second year.

The MoH’s increasing emphasis on chronic diseases and its special emphasis on diabetes are also reflected in the recent changes in the organization of the Ministry. As part of the HTP, the MoH was reorganized in November 3, 2011. All the departments of the MoH were abolished and new ones were established to meet the needs of the new health care system and changing trends in the healthcare environment. Within this new structuring, a new Directorate for Health Promotion was established reflecting the importance attributed to healthy life styles and health promotion in the future health policies.

Although these policy initiatives indicate a clear determination to prioritize diabetes management in the health care system, there are still caveats in implementation. It is not possible to state that the paradigm shift occurring at the policy level is also happening at the provider level as well. The health care providers are still very treatment oriented and this is fueled further by the current performance based payment system. Family practitioners are the main providers for implementation of these policies but lack of a referral system may hinder the attempts to use this professional force in the implementation of prevention-oriented policies. The introduction of additional incentives to the payment mechanism of family practitioners and rewarding their extra efforts may be useful in future.

As stated earlier, the SSI is the main reimbursement agency in Turkey and rules of reimbursement are determined through the Health Implementation Guide (HIG) published by the organization. In the latest available HIG, the rules for diabetes are presented in Table 3.

**Table 3:** Rules of reimbursement for diabetes treatment

**Treatment**	**Who can prescribe?**	**Patient restrictions**	**Other restrictions**
Metformin, Sulfonylurea, human insulin	All physicians	-	-
Repaglinide, nateglinide combined preparations of other oral anti-diabetics	Endocrinologists, internists, cardiologists and family practitioners (specialized)*	-	-
Analog insulin, pioglitazone, oral combinations of pioglitazone or combined use of pioglitazone with insulin	Endocrinologists, internists, cardiologists and family practitioners (specialized)*	-	-
DPP-4 antagonists (sitagliptine, vildagliptine) and combination of DPP-4 antagonists by other oral anti-diabetics	Endocrinologists and internists at tertiary level*	Only to patients without glycemic control after the use of maximum tolerable dose treatment by metformin and sulfonylurea	
Exanatide	Endocrinologists	• Patients without glycemic control with the use of metformin and sulfonylurea at maximum tolerable doses and with a body mass index over 35 kg/m2	• Cannot be used with insulin
			• Treatment stops after development of pancreatitis
		• Patients without history of pancreatitis	
Blood glucose meters and strips	• Endocrinologists, internists, or specialized family practitioners. *		

* These can be prescribed by all physicians after a medical report issued by one of these specialists.

## Priorities for the future

The current emphasis on diabetes and its risk factors marks the beginning of a new era in the Turkish health care policy environment. In the past, the treatment-oriented nature of the health care system had been criticized fiercely for not taking into account preventive services at the expense of curative services. The new organization of the MoH can also positively influence the implementation of new policies. However, only making policies or developing action plans is not enough to guarantee their implementation. There are a number of priorities for the future regarding the management of diabetes and its complications.

First, both making and implementing diabetes related policies require an evidence-based approach. This indicates the need for a better health information system. Available epidemiologic and other data are not sufficient to diagnose the real extent of the problem. The development of a diabetes registry could be the first step for the provision of necessary evidence. The government, academicians, non-governmental organizations, patients and health care providers should participate in the preparation of the information system as key stakeholders that will use the end products of this system.

Second, the cost of diabetes and diabetes related complications should be studied from the SSI and societal perspective. The methodology of these studies should be shared with the scientific community. The study should be designed to represent the whole country.

Third, national diagnosis and treatment guidelines should be developed with a wide participation from different professional areas. These guidelines must be endorsed by the SSI and should form the basis for reimbursement decisions by the social security system. Guidelines will also help to provide balanced diagnosis, treatment and monitoring options to the citizens as well.

Last but by no means the least, increasing awareness of the public should be a priority in order to diagnose and treat the disease at earlier stages. According to the TURDEP II study, 45% of the participants were not aware they had the disease. Given the fact that accessibility is better than the past, this figure shows other contributors have an impact on these figures. The family practitioners are of special importance in increasing this awareness. The family practitioners should be trained to detect and follow the risk factors of diabetes among their patients. Special training sessions can be organized at the primary level of care with special emphasis on increasing the awareness for diabetes. A important health workforce that is not used effectively, pharmacists, can also play a prominent role in decreasing the awareness figures for diabetes as well. There are more than 20 000 community pharmacists in Turkey scattered in all geographical regions of the country with easy access. The discussion to redefine the role of these pharmacists has already started in Turkey and the pharmacists are eager to widen their roles as dispensers of pharmaceuticals. This well educated and already available workforce can be used to increase the awareness levels of diabetes in the community without extra effort and cost.

## Abbreviations

BoD: Burden of disease; DALY: Disability adjusted life years; HIG: Health implementation guide; HTP: Health transformation program; IDF: International diabetes federation; MoH: Ministry of health; SSI: Social security institution; USD: US dollars.

## Competing interests

The author declared that they have no competing interest.
